# Stability of Domoic Acid in 50% Methanol Extracts and Raw Fecal Material from Bowhead Whales (*Balaena mysticetus*)

**DOI:** 10.3390/md19080423

**Published:** 2021-07-27

**Authors:** Emily K. Bowers, Raphaela Stimmelmayr, Kathi A. Lefebvre

**Affiliations:** 1 Northwest Fisheries Science Center, Environmental and Fisheries Sciences Division, National Marine Fisheries Service, NOAA, 2725 Montlake Blvd E, Seattle, WA 98112, USA; emily.bowers@noaa.gov; 2 The North Slope Borough Department of Wildlife Management, P.O. Box 69, Utqiagvik, AK 99723, USA; rafstimmel@gmail.com

**Keywords:** marine mammals, toxin degradation, harmful algal bloom toxins, storage conditions, ELISA, domoic acid

## Abstract

Domoic acid (DA), the toxin causing amnesic shellfish poisoning (ASP), is produced globally by some diatoms in the genus *Pseudo-nitzschia*. DA has been detected in several marine mammal species in the Alaskan Arctic, raising health concerns for marine mammals and subsistence communities dependent upon them. Gastrointestinal matrices are routinely used to detect Harmful Algal Bloom (HAB) toxin presence in marine mammals, yet DA stability has only been studied extensively in shellfish-related matrices. To address this knowledge gap, we quantified DA in bowhead whale fecal samples at multiple time points for two groups: (1) 50% methanol extracts from feces, and (2) raw feces stored in several conditions. DA concentrations decreased to 70 ± 7.1% of time zero (T_0_) in the 50% methanol extracts after 2 weeks, but remained steady until the final time point at 5 weeks (66 ± 5.7% T_0_). In contrast, DA concentrations were stable or increased in raw fecal material after 8 weeks of freezer storage (−20 °C), at room temperature (RT) in the dark, or refrigerated at 1 °C. DA concentrations in raw feces stored in an incubator (37 °C) or at RT in the light decreased to 77 ± 2.8% and 90 ± 15.0% T_0_ at 8 weeks, respectively. Evaporation during storage of raw fecal material is a likely cause of the increased DA concentrations observed over time with the highest increase to 126 ± 7.6% T_0_ after 3.2 years of frozen storage. These results provide valuable information for developing appropriate sample storage procedures for marine mammal fecal samples.

## 1. Introduction

Harmful algal blooms (HABs) create health concerns for humans and wildlife worldwide due to the production of potent toxins that can accumulate in filter-feeding organisms such as planktivorous fish, bivalves, krill, and other invertebrates [1,2,3,4,5]. These organisms act as vectors of HAB toxins to upper trophic level predators and can cause significant health impacts and mortality in fish, marine mammals, and seabirds [6,7,8,9,10,11,12]. HAB toxin risks to human seafood consumers have been mitigated by the implementation of seafood safety regulatory limits for allowable levels of toxins in seafood designated for human consumption [13]. There are growing concerns, however, regarding the apparent increase in HAB frequency, toxicity, and geographic and temporal distribution around the world, to which climate change, eutrophication, and ballast water transfer are contributing [3,14,15]. Changes in HAB distribution raise public health and wildlife management concerns, particularly for locations previously unexposed or poorly monitored for HABs, such as the expansive coastlines of Alaska [16,17].

Domoic acid (DA) is a common HAB toxin, produced by some diatoms in the genus *Pseudo-nitzschia* [18]. Acute exposure to DA causes the human neurological illness amnesic shellfish poisoning (ASP) [19]. Most commonly contracted by consuming shellfish that feed on toxic phytoplankton, ASP can be fatal with high levels of DA ingestion [9]. In marine mammals such as California sea lions (*Zalophus californianus*) and Northern fur seals (*Callorhinus ursinus*), DA causes symptoms such as seizures, ataxia, reduced responsiveness, and brain and heart lesions [20,21]. DA toxicosis can result in mortality, as was the case in the 1998 unusual mortality event (UME) of California sea lions along the central California coast [7,10]. Even sublethal exposure is cause for concern, as DA has been found to cause chronic neurological effects such as memory and learning impairment in sea lions (*Z. californianus*) [22]. Exposure to DA has been reported in humpback (*Megaptera novaeangliae*), blue (*Balaenoptera musculus*), minke (*B. acutorostrata*), pygmy (*Kogia breviceps*), dwarf sperm (*K. sima*), and North Atlantic right whales (*Eubalaena glacialis*) [5,23,24,25], Northern fur seals (*Callorhinus ursinus*) [21], harbor seals (*Phoca vitulina*) [26,27], Northern sea otters (*Enhydra lutris*) [28], and 13 Arctic marine mammal species including Pacific walruses (*Odobenus rosmarus*), bowhead whales (*Balaena mysticetus*), and ice seals (*Pusa hispida, Histriophoca fasciata, Erignathus barbatus, Phoca largha*) [17].

Marine mammals are important subsistence resources for Arctic and subarctic communities. Therefore, monitoring the impact of HAB toxins on Arctic marine mammal health is of critical importance for many local, state, federal, academic, and tribal institutions, nationally and internationally, that participate in research on Arctic wildlife and ecosystem health. Gastrointestinal (GI) contents have been the primary matrices in which HAB toxins are quantified for routine marine mammal monitoring and health assessments during UMEs; therefore, validating the reliability of quantifications from these matrices is of great importance [17].

Many studies have been conducted assessing the chemical and biological stability of DA in experimental settings and in matrices associated with seafood safety. We know that DA is stable at room temperature in saline solution [29], and that it degrades with sunlight exposure in seawater [30,31] and when stored at high temperatures and extreme pH in aqueous solution [32,33]. DA depurates in the bodies of living vector organisms at various rates, depending on the species [19], but is not degraded via steaming or autoclaving in harvested shellfish [34] and was even reported to become more concentrated in scallops stored at 12 °C [35]. However, little is known about the stability of DA in routinely-used marine mammal matrices such as GI contents. Toxin stability between sample collection and quantification is paramount, as it underpins all upstream analyses and results interpretation. Delayed sample collection and improper or prolonged storage are not uncommon for remote field sampling, making it imperative to quantify potential impacts of these factors on the accuracy of toxin measurements in GI contents from harvested, stranded, or dead marine mammals sampled in the field.

**Figure 1:**
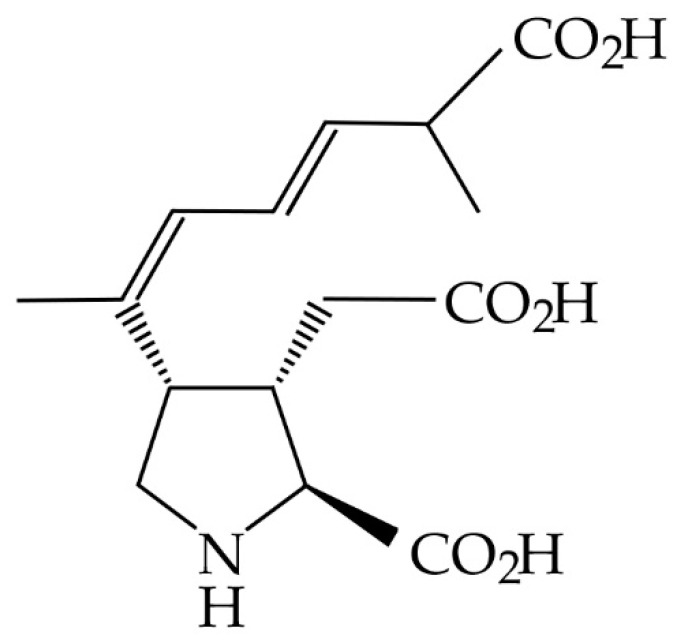
The chemical structure of domoic acid.

This study aims to characterize DA (Figure 1) stability in field-collected marine mammal feces and fecal extracts under various storage conditions. DA concentration in bowhead whale *(Balaena mysticetus*) feces was quantified at multiple timepoints for two groups: (1) 50% methanol extracts from feces (Extract group; stored at 1 °C in the refrigerator) and (2) raw feces (Raw Feces Treatment groups; stored at −20 °C in the freezer, 1 °C in the refrigerator, room temperature (RT) dark, RT light, or in an incubator at 37 °C). Toxin stability, as percent of initial concentration (%T_0_), is reported here for each study group.

## 2. Results & Discussion

### 2.1. Extract Group

**Figure 2:**
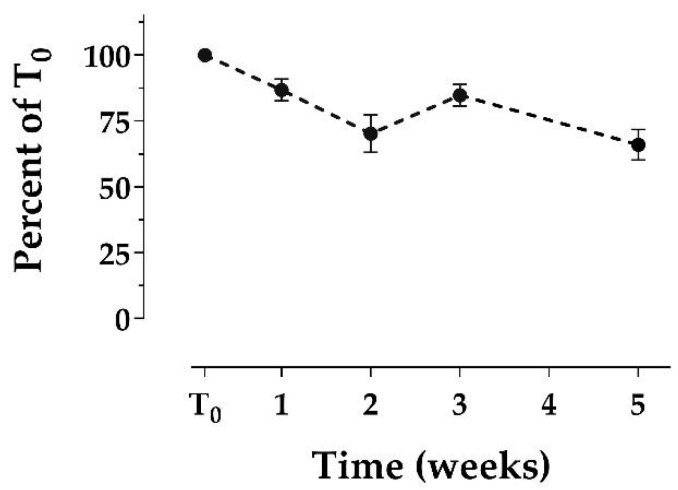
Stability of domoic acid in 50% methanol extracts from bowhead whale feces over time, reported as the average of the percent of time-zero concentration. Extracts were refrigerated at 1 °C in the dark. Each time point consists of the same *n* = 5 samples. Error bars display the standard error of the mean (SEM). Domoic acid concentrations ranged from 57 ng/g to 1161 ng/g, which were well above the minimum detection limit of 4 ng/g.

We extracted bowhead whale feces collected from five individual whales using 50% methanol, and extracts from each replicate were repeatedly analyzed via enzyme-linked immunosorbent assay (ELISA) over 5 weeks (see the Supplementary Materials). Extracts were refrigerated in the dark at 1 °C for the duration of the study. The average DA concentration (as %T_0_) declined to 87 ± 4.1% after 1 week, then to 70 ± 7.1% after 2 weeks (Figure 2). The %T_0_ does not appear to decline at the same rate after 2 weeks: the 5-week T_0_ (66 ± 5.7%) is comparable to the T_0_ after 2 weeks, with the %T_0_ at week 3 being slightly higher (85 ± 4.1%). The standard errors of the mean (SEMs) were relatively low at all time points (≤7.1%; Figure 2). While stability of fecal methanol extracts has not been reported to date apart from this study, degradation of shellfish methanol extracts over time has been reported or suggested in several other studies, to varying degrees [32,35,36,37]. Variability between reported trends (e.g., short-term, dramatic degradation in Smith et al. [35] versus slow degradation over months in Vale and Sampayo [37] versus the moderate degradation observed here) may be attributable to differences in methanol percentage, methanol-to-sample ratio, or sample matrix. Collectively, our Extract group results suggest that DA from marine mammal feces extracted in 50% methanol is subject to moderate degradation (~30%) in at least the first 2 weeks of refrigeration. To obtain the most accurate results, we recommend analyzing extracts as soon as possible (within 1 week of extraction).

### 2.2. Treatment Group

**Figure 3:**
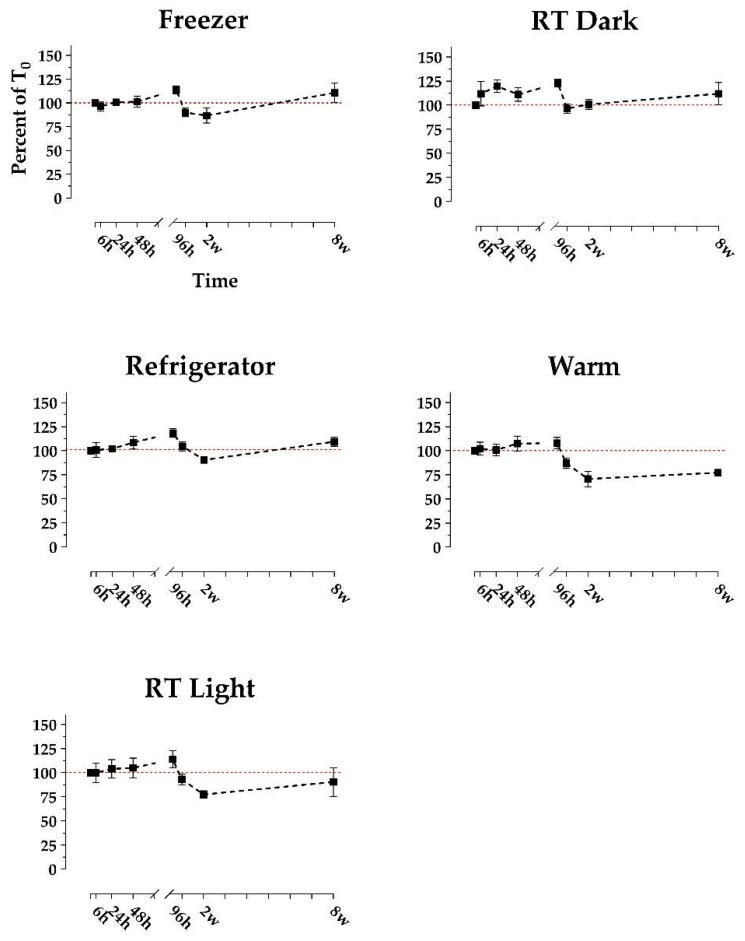
Stability of domoic acid in raw bowhead whale feces over time, reported as the average of the percent of time-zero concentration. Fecal aliquots were stored in 5 treatment conditions (freezer at −20 °C, refrigerator at 1 °C, room temperature (RT) dark, RT light, and incubator at 37 °C). Each time point consists of *n* = 4 samples. A 100% reference line is included in red. Error bars display the SEM. Domoic acid concentrations ranged from 67 ng/g to 9812 ng/g, which were well above the minimum detection limit of 4 ng/g.

**Figure 4:**
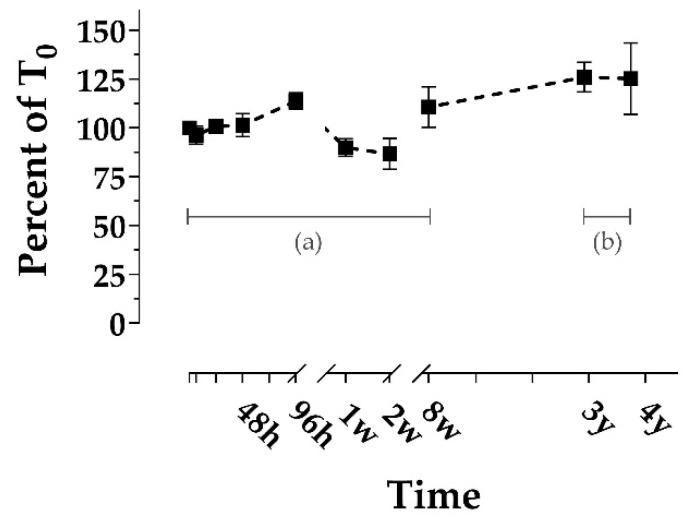
Stability of domoic acid in raw, frozen bowhead whale feces over time, reported as the average of the percent of time-zero concentration. Short-term time points (**a**) consist of the same *n* = 4 samples repeatedly analyzed (see Figure 1). Long-term time points (**b**) consist of separate *n* = 4 samples analyzed at 2 time points (3.2 and 3.8 years). Error bars display the SEM. Domoic acid concentrations ranged from 124 ng/g to 9812 ng/g, which were well above the minimum detection limit of 4 ng/g.

Replicates of *n* = 4 bowhead whale fecal samples collected from four individual whales were stored in five treatments: (1) freezer (−20 °C), (2) refrigerator (1 °C), (3) room temperature (RT) in the dark, (4) RT in the light, and (5) in an incubator (~37 °C). Sample aliquots were analyzed repeatedly to quantify DA concentrations via ELISA over 8 weeks. Additionally, we opportunistically analyzed *n* = 4 samples that were stored frozen for 3.2 and 3.8 years (long-term frozen). DA concentrations appeared reasonably consistent over time in 0–8-week (short-term) treatments (Figure 3), while the long-term frozen samples yielded consistent but slightly higher concentrations (Figure 4).

Average DA concentration (as %T_0_) remained between approximately 70 and 110% in all short-term treatments (Figure 3), while long-term frozen %T_0_ increased to 126 ± 7.6% and 125 ± 18.3% for the 3.2 and 3.8-year time points, respectively (Figure 4). The rise above 100% T_0_ in long-term frozen samples is likely attributable to moisture evaporation from within the sample, causing DA to concentrate. Smith et al. [35] also reported increased DA concentration in raw scallops stored at 12 °C for ≤3 days, likewise implicating evaporation. Additionally, the long-term increases could be due at least in part to variability between ELISA kits and days. While the RT Light and Warm samples appeared to decline relatively rapidly between 96 h and 2 weeks, this observation is not sufficiently distinct to draw qualitative conclusions (Figure 3). However, across the Freezer, Refrigerator, and RT Dark treatments, we did notice slight increases at the 96-h mark, as well as a slight increase at the 8-week mark in the Freezer and Refrigerator treatments (Figure 3). This implicates an artifact introduced by the ELISA plates or the standards with which time points are analyzed. The SEM was relatively low for all short-term time points and treatments but higher for the 3.8-year frozen time point (Figure 4). The higher 3.8-year SEM is likely attributed to moisture evaporation occurring to varying degrees over time between replicates.

Altogether, we observe no empirically obvious reductions in DA concentration in any treatment. This suggests that DA is robust against degradation in marine mammal feces in a surprising variety of conditions, and that concerns of significantly underestimated values need not necessarily be raised based on sample storage conditions of ≤8 weeks. The moderate increases observed in long-term frozen concentrations (~25% above T_0_) were well within an order of magnitude of T_0_. However, care should be taken to minimize sample evaporation and analyze samples promptly whenever possible to yield the most accurate results. Inflating DA concentrations by 25% may be a reasonable adjustment for samples from which evaporation is suspected.

## 3. Materials & Methods

### 3.1. Sample Collection and Selection

During 2016–2019, fecal samples from subsistence-harvested bowhead whales were collected in Utqiagvik, Alaska. Sections of colon were cut and fecal matter was removed using plastic spoons. Fecal samples were collected in 50-mL polypropylene screw-cap tubes (Falcon-BD, Franklin Lakes, NJ, USA) and stored frozen at −20 °C until time of analysis. Samples were shipped to the Northwest Fisheries Science Center’s Wildlife Algal-Toxin Research and Response Network (WARRN-West) laboratory (Seattle, WA, USA) for toxin analysis.

All samples were originally subsampled and analyzed for DA 2–8 months after sample collection. Remaining unanalyzed sample material was kept frozen. We selected samples for the present study based on their original DA concentration and date of analysis, choosing samples with original concentrations of >100 ng/g whenever possible.

### 3.2. Study Setup

The goal of the Extract group was to characterize DA stability in 50% methanol extracts from marine mammal feces stored in the cold and dark. We extracted raw bowhead whale feces (*n* = 5) following the extraction procedure outlined for the Extract group below (3.3.1). Extracts were stored in a Kenmore top-mount refrigerator (model no. 253.68972802).

The goal of the Treatment group was to characterize DA stability over time in raw marine mammal feces under various relevant conditions. Fecal aliquots from *n* = 4 bowhead whales were stored under 5 treatments: freezer, refrigerator, room temperature (RT) dark, RT light, and warm incubator (Table 1).

During the treatment period, freezer samples were stored in a Frigidaire freezer (model FFFU21M1QWE), and refrigerator samples were stored in the refrigerator specified above. All RT samples were kept on a North-facing window sill, and RT light samples received natural (but not direct) sunlight during daylight hours in Seattle, WA, USA (December–February). RT light aliquot tubes were lined up on wire racks that allowed full light exposure, while RT dark samples were kept under a light-proof box. Ambient temperature was monitored for the RT samples using a TP-50 digital air thermometer (ThermoPro, Toronto, ON, Canada). Warm samples were kept in a Lab-Line (model no. 120) incubator, and internal incubator temperature was monitored using a Fisher USA thermometer (90 mm, Waltham, MA USA).

Additional raw fecal samples were also analyzed after two durations of freezer storage (up to 3.8 years) to assess long-term DA stability under standard storage conditions (−20 °C; see freezer specifications above). Along with the four samples used in the 8-week short-term treatment study described above, we analyzed four additional fecal samples at two time points each (3.2 and 3.8 years of frozen storage).

### 3.3. Toxin Extraction

#### 3.3.1. Extract Group

We extracted DA via methanol dilution, homogenization, and centrifugation. Raw fecal samples were thawed at room temperature and stirred thoroughly. For each sample, we weighed out approximately 1 g of fecal material (Scout STX balance, Ohaus, Parsippany, NJ, USA) and aliquoted it into a 14-mL polypropylene pop-cap tube (Falcon-BD, Franklin Lakes, NJ, USA). We added 50% methanol (the standard extraction solvent for DA ELISA analyses) to each aliquot at 3× the aliquot weight for a 1-in-4 dilution. Sample solutions were briefly vortexed on high (Analogue Vortex Mixer, sn 060223013, VWR, Radnor, PA, USA), then homogenized with a generator probe (GLH 850, Omni-International, Kennesaw, GA, USA) for 1 min at 2100 rpm. Homogenized samples were then centrifuged at 5000 rpm for 20 min at 4 °C (CR3i centrifuge, Jouan, Milford, MA, USA). Supernatants (the extracts) were poured into 4-mL glass amber vials (National C4015-2W Thermo Scientific, Waltham, MA USA) and refrigerated until further analysis. Directly prior to toxin quantification, we filtered 200 μg subsamples of the extracts for analysis (Ultra-Free Centrifugal filters, 0.22 μm, UFC30GVNB, Millipore Sigma, Chicago, IL, USA). Extracts were sampled, filtered, and re-analyzed at each time point.

#### 3.3.2. Treatment Group

Toxin extraction for the raw, short-term Treatment samples is modified from the Extract group extraction described above. Samples were thawed in a refrigerator for 4 h, stirred, and aliquoted. From each sample, we weighed out five 3-g aliquots (one per treatment) and stored them in 14-mL tubes. We extracted a base time point (T_0_) from each aliquot directly prior to beginning treatments. At each time point, the aliquots in 14-mL tubes were again stirred (except the frozen treatment aliquots). We then aliquoted 0.25 g of feces from each 14-mL tube into 1.5-mL plastic microfuge tubes on ice (frozen treatment aliquots were scraped from the top of the still-frozen samples). Light exposure was minimized for all treatment groups during aliquoting except for the RT light treatment. After aliquoting, we added 50% methanol to the 1.5-mL microfuge tubes as described above. Fecal-methanol solutions were vortexed on high for three 30-s increments (90 s total; see above vortexer information), being held in the refrigerator for approximately 2 min between increments to keep cool. After 90 s of vortexing, samples with remaining fecal clumps were vortexed for additional time and/or clumps were broken using metal spatulas until samples were of uniform consistency. Finally, we incubated the fecal-methanol slurries in the freezer for 5 min, then centrifuged them (accuSpin Micro 17 Fisher Scientific, Waltham, MA USA) for 12 min at 12,000 rpm. Supernatant extracts were then poured off into clear 1.5-mL cryovials (220-3902-080, Evergreen Scientific, Caplugs CA, USA) and refrigerated until further analysis (mean 2.5 days). Directly prior to toxin quantification, extracts were filtered as described above. Aliquoting from 14-mL tubes and extraction was repeated at each time point; extracts were not re-analyzed. Long-term frozen treatment samples were aliquoted and extracted according to the procedure outlined for the Extract group (3.3.1), except long-term frozen samples were re-aliquoted and extracted at each time point (each extract was analyzed only once).

### 3.4. Toxin Quantification

We quantified DA via direct-competition ELISA using commercially-available Biosense^®^ ASP ELISA kits (Biosense^®^ Laboratories, Bergen, Norway). While these kits are intended for analyzing shellfish and water samples, previous studies by our team were performed to determine appropriate dilutions to avoid matrix effects from marine mammal matrices, and for for validation of other analytical methods compared to ELISA results [21,38]. Kits were used according to their manufacturer protocol with dilution modifications from Frame and Lefebvre [38] (base dilution of 1:100 sample: 50% methanol, plus any additional dilution necessary to ensure each concentration falls within the kit’s working range. Final dilutions were determined individually upon original analysis prior to this present study and remained constant throughout the study). Kit plates loaded with samples, standards, and reagents were incubated at room temperature on an orbital shaker (Bellco Biotechnology, Vineland, NJ, USA) for 75 min, then washed (ELx50, sn 257474, BioTek, Winooski, VT, USA). Kit-provided color solution was added to all wells before plates were incubated again for 15 min on the orbital shaker. Well absorbance was quantified using a BioTek Epoch (sn 257814). The detection limit for fecal samples by ELISA was 4 ng/g. Domoic acid concentrations quantified in all samples used in this study ranged from 57 ng/g to 9812 ng/g.

### 3.5. Data Analysis

We interpolated unknown DA concentrations using known standard absorbances and concentrations with the 4-parameter logistic curve fit model recommended in the Biosense^®^ protocols. Resulting concentrations were reported as percentages of the respective initial concentrations, T_0_ ([T_i_/T_0_] × 100). For the Extract and short-term Treatment groups, we defined T_0_ as the DA concentration quantified at the beginning of the present study, controlling for any changes that may have occurred after original analyses in the past and the present study. Conversely, the purpose of the long-term frozen samples was to assess DA stability in the years following original sample analysis; hence, T_0_ for long-term frozen samples was defined as the original concentration quantified years prior.

No statistical analyses were used to evaluate this present study due to small sample sizes (*n* ≤ 5 replicates) which limited the reliability of data normality assessments and any subsequent parametric or non-parametric analyses [39,40,41]. Sample size was constrained in this study by sample availability, sample volume, and original toxin concentration.

## 4. Conclusions

This study aimed to characterize DA stability in extracted and raw marine mammal fecal material to assess the risk of toxin degradation due to storage time and conditions when quantifying DA in field-collected marine mammal GI samples for wildlife health assessments. This study provides evidence for DA being stable in raw bowhead whale feces frozen at −20 °C as well as stored in a refrigerator and at room temperature in the dark, with only slight toxin loss observed in the RT light treatment and moderate toxin loss in the 37 °C incubator treatment with a storage period up to 8 weeks. Most notable are the findings that DA concentrations were not significantly reduced during longer term frozen storage up to 3.8 years, and in fact, that increases were observed, likely due to evaporative processes. DA appeared less stable over time in 50% methanol extracts, decreasing to approximately 70% of T_0_ in the first two weeks and suggesting that 50% methanol extracts should be analyzed as promptly as possible. In addition to evaporative processes, inherent differences between plates and the execution of analyses may contribute to variability in toxin quantifications. However, the ELISA protocol calls for fresh standards to be prepared with each plate, thereby reducing any quantitative differences from plate and analyst performance between assays. Further studies are necessary to confirm the generalizability of these results; however, we expect results to be similar for other marine mammal species. Also, given that feces is one of the “messiest” marine mammal matrices, requiring a higher dilution than most marine mammal matrices to counter matrix effects [38], we anticipate other matrices will display similar (or greater) consistency in toxin concentration over time.

## Figures and Tables

**Table 1:** Summary of Treatment group design and justification.

Treatment Name	Temperature	Justification
Freezer	−20 °C	Ideal condition in which samples are stored immediately upon collection
Refrigerator	1 °C	Second best storage option if freezing is not possible
Room Temperature (RT)—Dark	18 °C ± 2 °C ^1^	Accidental/unavoidable exposure to ambient temperatures (e.g., field, laboratory) in the dark
Room Temperature (RT)—Light	18 °C ± 2 °C ^1^	Accidental/unavoidable exposure to ambient temperatures (e.g., field, laboratory) in daylight
Warm	35 °C ± 3 °C ^1^	Delay of sample collection from a dead carcass

^1^ Mean temperature ± standard deviation.

## Data Availability

The data presented in this study are available as Supplementary Material published with this manuscript.

## Supplementary Materials

The following are available online at https://www.mdpi.com/article/10.3390/md19080423/s1.
